# Phylogenetic analyses of complete mitochondrial genome sequences suggest a basal divergence of the enigmatic rodent *Anomalurus*

**DOI:** 10.1186/1471-2148-7-16

**Published:** 2007-02-08

**Authors:** David S Horner, Konstantinos Lefkimmiatis, Aurelio Reyes, Carmela Gissi, Cecilia Saccone, Graziano Pesole

**Affiliations:** 1 Dipartimento di Scienze Biomolecolari e Biotecnologie, Università di Milano, Via Celoria 26, 20133 Milano, Italy; 2 Istituto Tecnologie Biomediche, Consiglio Nazionale delle Ricerche, via Amendola 122/D, 70125 Bari, Italy; 3 Dipartimento di Biochimica e Biologia Molecolare, Università di Bari, Via Orabona 4, 70126 Bari, Italy; 4 Harvard Medical School, West Roxbury VAMC, West Roxbury, MA 02132, USA; 5 MRC Dunn Human Nutrition Unit, Wellcome Trust/MRC Building, Hills Road, Cambridge CB2 0XY, UK

## Abstract

**Background:**

Phylogenetic relationships between Lagomorpha, Rodentia and Primates and their allies (Euarchontoglires) have long been debated. While it is now generally agreed that Rodentia constitutes a monophyletic sister-group of Lagomorpha and that this clade (Glires) is sister to Primates and Dermoptera, higher-level relationships within Rodentia remain contentious.

**Results:**

We have sequenced and performed extensive evolutionary analyses on the mitochondrial genome of the scaly-tailed flying squirrel *Anomalurus sp*., an enigmatic rodent whose phylogenetic affinities have been obscure and extensively debated. Our phylogenetic analyses of the coding regions of available complete mitochondrial genome sequences from Euarchontoglires suggest that *Anomalurus *is a sister taxon to the Hystricognathi, and that this clade represents the most basal divergence among sampled Rodentia. Bayesian dating methods incorporating a relaxed molecular clock provide divergence-time estimates which are consistently in agreement with the fossil record and which indicate a rapid radiation within Glires around 60 million years ago.

**Conclusion:**

Taken together, the data presented provide a working hypothesis as to the phylogenetic placement of *Anomalurus*, underline the utility of mitochondrial sequences in the resolution of even relatively deep divergences and go some way to explaining the difficulty of conclusively resolving higher-level relationships within Glires with available data and methodologies.

## Background

Phylogenetic relationships and divergence times within the superorder Glires (Rodentia, and Lagomorpha) remain controversial, with many discrepancies between estimates from morphological, molecular and fossil data. The problem is exacerbated both by the fact that Rodentia represents the most abundant and diversified order of living mammals and by variations in molecular evolutionary rate and mode in some families. For example, several molecular studies have suggested paraphyly of Rodentia or Glires [1-3], while others (and the majority of morphological data) support the monophyly of both groups [4-6]. Both molecular approaches and morphological analyses have their limitations. Critics of conclusions based on molecular characters cite the limited number of sequences considered and the apparent dependence of conclusions on the analytical methodologies employed, while adherents of molecular data point out that the predominantly dental and cranial characters employed in morphological analyses are likely subject to homoplastic evolution as a result of shared ecological constraints. Some intra-ordinal phylogenetic relationships in Rodentia also remain poorly resolved. For example, while the monophyly of many classically diagnosed Rodentia groups (Hystricognathi – a grouping of Myoxidae and Sciuridae – and the Muroidea/Dipodidae group) have been supported by molecular analyses (eg [4,7]), relationships between these groups as well as the placement of a few under-studied taxa (such as the Anomaluridae) are controversial. Discrepancies between molecular and other data are not restricted to tree topologies. Molecular dating approaches (typically employing mitochondrial DNA sequences) have tended to provide estimates of divergence times which conflict with inferences drawn from the fossil record. More recently the availability of relaxed and local molecular clock approaches [8], which allow evolutionary rates to differ across the tree, has allowed some reconciliation of molecular and fossil derived divergence time estimates within Euarchontoglires [9,10].

In the current study, we have sequenced and analysed the complete mitochondrial genome of *Anomalurus sp*. as a representative of the Anomaluridae, a family of flying squirrel-like rodents which possess two rows of pointed, raised scales on the undersides of their tails and whose cranial anatomy does not indicate a close relationship with sciurid flying squirrels. Indeed, the phylogenetic affinities of the Anomaluridae, which consists of three extant genera and whose geographic distribution is currently restricted to central Africa, have remained enigmatic owing both to the aforementioned weakness of morphological characters in the systematics of Rodentia and a relative lack of available molecular sequence data (currently restricted to five nuclear and two mitochondrial gene sequences). Previous studies based on molecular data have suggested alternative phylogenetic placements for *Anomalurus*, while weakly supporting various relationships between the Hystricognathi, the Sciuridae, and the Muroidea/Dipodidae group [11-13], while morphological classifications have suggested almost all possible placements for *Anomalurus *(reviewed in [14]).

We have performed extensive phylogenetic analyses of the protein coding regions of all available Primates, Lagomorpha and Rodentia mitochondrial genomes at both nucleotide and inferred amino acid sequence levels. We show that the sequence data suggest a phylogenetic affinity between *Anomalurus *and the Hystricognathi. However, statistical tests of alternative tree topologies do not exclude other phylogenetic hypotheses, either for the placement of *Anomalurus sp*. or for higher-level relationships within Rodentia. These observations are at least partially explained by a Bayesian relaxed molecular dating approach which generates estimates of divergence times within Euarchontoglires that are compatible with fossil and biogeographical data and suggest that a rapid evolutionary radiation within Glires occurred around 60 million years ago.

## Results

### The mitochondrial genome of *Anomalurus*

The mtDNA of *Anomalurus *is 16,923 bp long and presents the common vertebrate gene organization. The entire genome sequence has been submitted to the EMBL sequence database under accession number AM_159537. Start and end positions of all protein coding, tRNA and rRNA genes were easily identifiable through homology searches using characterized mammalian mitochondrial protein sequences as probes. The control region (D-loop containing region) is 1439 bp long and shows the typical tripartite structure observed in mammals with the central conserved domain (15,770–16,062) and the CSB domain (16,063–16,923) both identifiable. Of the two conserved blocks known to be located in the ETAS domain, ETAS1 and ETAS2 [15], only a 40 bp long conserved sequence corresponding to ETAS1 can be identified (15641–15681). Indeed, only this element is conserved across Rodentia [16]. The CSB domain includes all the three known conserved sequence blocks (CSB1, CSB2 and CSB3), and contains a tandem repeat array made up of a 40-fold repetition of an 8 bp long monomer (CGTACAGC).

### Phylogenetic analyses

While a concatenated dataset of unambiguously aligned regions of H-strand protein sequences (all protein-coding genes apart from NAD6) passed the compositional homogeneity test implemented in TREE-PUZZLE [17], many corresponding DNA sequences failed the equivalent test. Compositional heterogeneity was reduced by the removal of third codon positions from the DNA dataset, although several sequences still failed the chi square test. We have previously shown that first position synonymous leucine codon usage (Leu-SynP1) varies extensively between mitochondrial genomes and is a source of compositional heterogeneity [6]. Accordingly, we removed (Leu-SynP1) codons from the alignment resulting in a dataset where only sequences from the Cercopithecinae (*Papio*, *Macaca*, *Chlorocebus*) failed the test of compositional homogeneity. Bearing this result in mind, phylogenetic analyses at the DNA level were performed both in the presence and absence of sequences from Primates.

**Figure 1:**
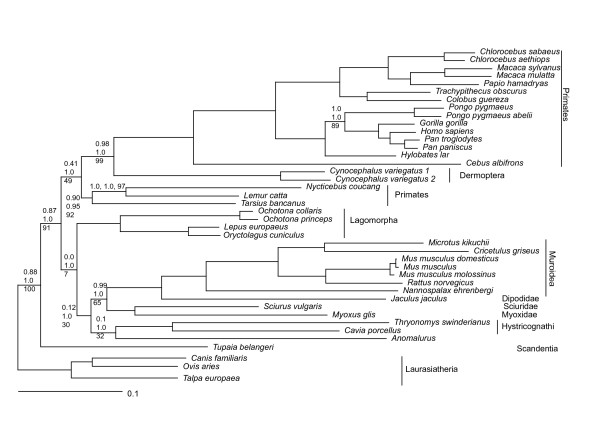
**Relationships within Euarchontoglires inferred from Bayesian analysis of 3519 unambiguously aligned amino acids encoded by H-strand mitochondrial genes**. The tree was recovered under the mtREV amino acid model with invariable and 8 gamma distributed variable rate categories. Bayesian posterior probabilities (nucleotide analyses), Bayesian posterior probabilities (amino acid analyses) and distance bootstrap (amino acid analyses, under the same model used for Bayesian analyses) are shown where any of these support values were not 100%.

Fig. 1 shows the Bayesian consensus tree of Euarchontoglires relationships inferred from protein sequences (with Bayesian Posterior Probabilities (PP) and distance bootstrap (BP) values associated with branches). Relationships recovered within Anthropoidea all have high bootstrap and posterior support and are uncontroversial. However, Primates emerge as a paraphyletic group with a clade defined by *Tarsius*, *Nycticebus *and *Lemur *diverging before the Dermoptera. Both the placement of *Tarsius *as sister to *Nycticebus *and *Lemur*, and the paraphyly of primates have been observed in other analyses of mitochondrial sequences (e.g. [18,6]). Scandentia (represented by the tree-shrew *Tupaia*) emerge basal to the Primates – Glires split. Given the controversy surrounding apparent discrepancies between mitochondrial and nuclear data with respect to these relationships, we conducted Approximately Unbiased (AU) tests [19] of competing topologies representing all plausible inter-relationships of Primates, Dermoptera, and Scandentia. At the 5% confidence level, the protein data exclude monophyletic primates regardless of whether *Tarsius *is sister to Anthropoidea or to *Lemur *and *Nycticebus*, while the DNA data permit monophyly of primates with Tarsius placed as sister to Anthropoidea (P = 0.059) or to *Lemur *and *Nycticebus *(P = 0.178). The protein (P = 0.062), but not the DNA sequences allow Scandentia to emerge immediately basal to Primates/Dermoptera (see discussion) but both datasets exclude a sister relationship between Scandentia and Dermoptera.

Notably, and in accord with our previous analyses [6], monophyly of Rodentia is supported with high Bayesian posterior support for protein-based analyses. However, protein distance-based bootstrap support for this partition is low (30%). Inspection of bootstrap partitions reveals that decay in support of Rodentia monophyly is caused by the sequence of *Anomalurus *and to a lesser extent those of *Thryonomys *and *Cavia *(Hystricognathi) that have a tendency to cluster with the outgroup sequences. Likewise, the monophyly of Glires receives high posterior support but does not emerge on the bootstrap consensus tree, owing to a tendency of Lagomorpha to emerge basal to the Rodentia/(Primates + Dermoptera) divergence in some bootstrap datasets. In accordance with other molecular studies [11,12], Bayesian analyses of protein sequences strongly support the monophyly of Hystricognathi, the monophyly of Myoxidae and Sciuridae and the monophyly of the Muroidea/Dipodidae grouping (all with BP = 100, PP = 1.0). In both Bayesian and distance bootstrap trees, *Anomalurus *emerges as sister of the Hystricognathi. Both methods suggest that the Hystricognathi/*Anomalurus *group is sister to a clade composed of Myoxidae/Sciuridae and the Muroidea/Dipodidae cluster. However, both the position of *Anomalurus *and the interrelationships between super-families within Rodentia receive only moderate posterior or bootstrap support. Bayesian analysis of the DNA data in the presence of the Cercopithecinae sequences yielded an identical tree topology apart from the position of *Anomalurus *which emerged as a poorly supported basal branch in the Primates/Dermoptera clade while Bayesian analyses of Glires, Scandentia and outgroup sequences alone generated an identical topology for Glires as the protein sequences (not shown).

In order to evaluate the degree of support for alternative hypotheses of relationships between Rodentia superfamilies, we generated a series of topologies where the constitution and internal topology of uncontroversial clades (outgroup, Primates/Dermoptera, Lagomorpha, Muroidea/Dipodidae, Myoxidae/Sciuridae and Hystricognathi) were constrained, but where inter-relationships of these groups and the placement of *Anomalurus *were varied. These topologies were tested under the AU test of alternative tree topologies. Selected results are shown in Table 1. In brief, both the protein and DNA data reject (at the 5% level) all topologies where *Anomalurus *is placed as sister to the Myoxidae/Sciuridae group, while all topologies depicting *Anomalurus *as sister to either Hystricognathi or the Muroidea/Dipodidae grouping are accepted as are various topologies placing *Anomalurus *either as the basal divergence among Rodentia or as sister to clades of Hystricognathi + Muroidea/Dipodidae, Hystricognathi + Myoxidae/Sciuridae or Muroidea/Dipodidae + Myoxidae/Sciuridae. Rodentia monophyly is moderately supported in that we were unable to identify acceptable topologies depicting Rodentia as non-monophyletic apart from those suggested by Bayesian analyses of the DNA data (*Anomalurus *as a basal divergence among Primates) – topologies where *Anomalurus *emerges as the basal branch of Glires were rejected by the protein data (P = 0.048) but were accepted by the DNA data. However, many topologies depicting non-monophyly of Glires (Lagomorpha divergence prior to the Rodentia/(Primate/Dermoptera) split, or Lagomorpha as sister to *Tupaia *– as suggested by other authors using mitochondrial sequence data [20,21]) were accepted by the AU test.

**Table 1:** Approximately Unbiased tests of selected alternative phylogenetic hypotheses of relationships within Euarchontoglires

	DNA		Protein	
		
Topology	Δ lnl	Pvalue	Δ lnl	Pvalue
1) Bayesian (protein) tree	5.7	0.579	BEST	0.771
2) Bayesian (DNA12) tree: (Glires,(*Anomalurus*/Primates/Dermoptera))	BEST	0.726	16.3	0.255
3) (Hystricognathi,((Dipodidae,Muroidea),(*Anomalurus*, (*Sciurus*,*Glis*))))	19.9	0.003*	21.2	0.007*
4) (Hystricognathi,((*Sciurus,Glis*),(*Anomalurus*, (Dipodidae,Muroidea))))	17.0	0.084	9.5	0.106
5) (Hystricognathi,(*Sciurus*,*Glis*)),(*Anomalurus*, (Dipodidae,Muroidea))))	15.4	0.169	1.4	0.623
6) *Anomalurus *basal in Rodentia	6.6	0.344	13.2	0.080
7) *Anomalurus *basal in Glires	8.7	0.120	20.4	0.048*
8) ((*Sciurus*,*Glis*),((Hystricognathi,*Anomalurus*), (Dipodidae,Muroidea)))	17.5	0.051	6.1	0.284
9) ((*Sciurus*,*Glis*),(Hystricognathi,(*Anomalurus*, (Dipodidae,Muroidea))))	17.6	0.104	7.0	0.294
10) (Lagomorpha,(Rodentia,Primates))	3.8	0.533	5.0	0.402

In all cases, the local topology of clades found in the Bayesian tree of protein sequences was retained and interrelationships between the sequences/clades specified were rearranged. Branchlengths and site likelihoods were optimized using PAML and the AU test implemented in the software CONSEL was applied. Topologies excluded by the AU test are marked with an asterisk.

### Slow-fast method

Given the apparent lack of resolution of Rodentia infra-ordinal relationships afforded by the mitochondrial sequence data and the tendency of the *Anomalurus *sequence to emerge in unexpected positions, especially for the DNA data, we wished to investigate whether undetected compositional or other types of systematic (or stochastic) biases manifested in faster evolving sites should be responsible for apparent decay in the phylogenetic signal. Accordingly we have used a variation on the "Slow-Fast" phylogenetic analysis methodology [22] where faster evolving sites are progressively removed from the protein alignment and bootstrap partitions recalculated. In the presence of misleading signal derived from homoplasy or biases at fast evolving sites, we might expect support for correct basal splits to increase as signal from slower evolving sites begins to predominate. We have used the SiteVarProt methodology [23] to estimate site-specific relative amino-acid substitution rates. We removed the fastest evolving 5% or 25% of sites (beyond this level, the preponderance of constant and autapomorphic sites tends to lead to the generation of very poorly resolved and supported topologies). Distance bootstrap analyses were performed on these datasets and support for key partitions was compared with support derived from the complete dataset (see Table 2). The set with the 5% of fastest evolving sites removed generated an identical bootstrap topology to the full set with comparable (+/- < 10%) bootstrap support values at all nodes with respect to the original data. However, when the most variable 25% of sites were removed, *Anomalurus *was recovered as sister to the Muroidea/Dipodidae clade, albeit with low (30%) bootstrap support, while bootstrap support for the partition *Anomalurus*+Hystricognathi fell to 28%. Additionally, support for Muroidea+Dipodidae+Sciuridae+Myoxidae fell to 13% while Hystricognathi emerged as sister to the *Anomalurus*/Muroidea/Dipodidae clade (23% BP, not shown in Table 2). Strikingly, and in accordance with the AU tests, *Anomalurus *never emerged as monophyletic with Sciuridae and Myoxidae, regardless of which set of sites were analysed.

**Table 2:** Distance bootstrap support (BS) for selected branches with indicated percentage of fastest evolving amino acid sites removed.

Partition	BS – 0%	BS – 5%	BS – 25%
(*Mus*)+*Rattus*	100	100	100
Muroidea+Dipodidae	100	100	97
Muroidea+Dipodidae+Sciuridae+Myoxidae	65	39	13
Hystricognathi+*Anomalurus*	30	32	28
*Anomalurus*+Dipodidae+Muroidea	4	4	30
*Anomalurus*+Scuridae+Myoxidae	0	0	0
*Chlorocebus*+*Macaca*+*Papio*	100	100	100
*Gorilla*+*Pan*+*Homo*	100	100	100

### Molecular dating

We employed the Bayesian protein phylogeny for use in a Bayesian relaxed clock dating approach [24] to estimate divergence times between major lineages. Relaxed clock methods allow substitution rates to vary over the tree and thus do not rely on strict clock-like evolution of the sequences under consideration. Following the method of Amer and Kumazawa [25] we have incorporated the mtREV24 + gamma model into the MULTIDISTRIBUTE software in order to use a substitution model developed with mitochondrial protein sequences. We also estimated divergence times using the DNA codon position 1 and 2 data under the F84 + gamma substitution model. For calibration points we specified that: 1) the Rodentia/Lagomorpha divergence should have occurred between 61 and 90 Million Years Ago (MYA) [26,27], 2) the basal divergence in the sampled Lagomorpha should have occurred between 35 and 40 MYA [28] and 3) the divergence of *Pongo *should have occurred between 13 and 18 MYA [29]. The inferred times of some key divergences (with associated errors) are shown in Table 3. Notably the divergence of Glires from Primates and Dermoptera is estimated to have occurred just over 65 Million Years Ago (MYA) by both DNA and protein data, the divergence of the Hystricognathi + *Anomalurus *group is dated at 58.7MYA (protein) or 57.5MYA (DNA), while the divergence of the Muroidea+Dipodidae clade from the Sciuridae+Myoxidae clade is estimated to have occurred 53.4MYA (protein) or 51.8 MYA (DNA). Importantly, nodes which were not used as calibration points were consistently dated in accordance with estimates from nuclear genes and the fossil record (Old world monkeys from New world monkeys 36.8MYA (protein) 38.4MYA (DNA), *Homo *from *Pan *5.7MYA (protein) 6.4MYA (DNA), *Mus *from *Rattus *15.1MYA (protein) 15.9MYA (DNA)) [13,30]. The estimates presented were generated using prior assumptions that the mean and standard error of the probability distribution describing the substitution rate at the root of the tree (a parameter required by the MULTIDIVTIME software) were equal to the mean of the substitution rate over the tree (assuming that Euarchontoglires is 75 million years old). However, the results of the Bayesian dating were extremely robust to the value specified for this parameter. Repeated runs with differing values yielded extremely similar estimates of divergence times (not shown).

**Table 3:** Selected estimates of divergence dates and amino acid substitution rates in branches leading to the labelled divergence in Euarchontoglires.

	Calibration point	protein		DNA12		aa substitution rate
				
Divergence		time/MYA	5% interval/MYA	time/MYA	5% interval/MYA	
*Homo*		5.73	4.11 – 7.76	6.41	4.39 – 8.76	0.26
*Gorilla*		9.00	7.00 – 11.44	10.16	7.73 – 12.74	0.26
(*Pongo*)	**13–18 MYA**	15.20	13.13 – 17.75	15.84	13.23 – 17.90	0.30
old world/new		36.80	31.86 – 42.12	38.50	32.58 – 42.81	0.35
world monkeys						
*(Cynocephalus)*		49.34	44.34 – 54.77	52.81	46.83 – 59.14	0.24
*(Tarsius*,(*Nycticebus*+*Lemur*))		60.65	56.72 – 65.96	59.74	54.90 – 65.62	0.15
(Lagomorpha)	**61–90 MYA**	62.77	61.05 – 67.53	62.68	61.04 – 67.27	0.12
(Glires)		65.42	62.32 – 70.99	65.28	61.87 – 70.97	0.13
basal Lagomorpha divergence	**35–40 MYA**	38.94	36.33 – 39.97	38.85	36.05 – 39.97	0.10
(*Anomalurus*, Hystricognathi)		58.83	55.51 – 63.56	57.50	53.33 – 62.40	0.14
(Hystricognathi)		48.12	42.51 – 53.52	52.28	46.20 – 57.91	0.25
(Muroidea,Dipodoidea)		53.57	49.10 – 58.34	51.82	46.08 – 57.28	0.17
(*Mus*)		15.06	10.54 – 20.45	15.93	10.38 – 22.84	0.23
(Muridae)		36.11	29.87 – 42.23	36.48	29.24 – 43.44	0.26

### Evolutionary rates

The Bayesian dating analysis also permits estimates of variation in evolutionary rates across the tree. Evolutionary rates of proteins estimated for branches leading to some nodes of interest are shown in Table 3. Like the estimates of divergence dates, the rate estimates were rather robust to the parameterization of the distribution of evolutionary rates at the base of the tree. The amino acid substitution rate inferred for the divergence between Primates/Dermoptera and Glires 0.13%/MY) remains relatively constant until the divergence of the *Tarsius*+*Nycticebus*+*Lemur *clade (0.15%/MY), wherein a sharp rise in substitution rates is observed (0.24%/MY at the divergence of *Cynocephalus *and 0.35%/MY at the divergence between old world and new world monkeys. Rates remain high (or continue to increase) within the old world monkeys, but within the Hominoidea there is a notable decrease in substitution rates (0.26%/MY at the divergences both of *Homo *and *Gorilla*. With respect to the Glires, there is a slight tendency to increased amino acid substitution rates in the Muroidea, Hystricognathi and *Anomalurus *(0.23–0.26%/MY), while rates remain relatively stable in Lagomorpha and *Sciurus*. We wished to investigate whether the observed lineage specific shifts in amino acid substitution rates were a general property of mitochondrial protein coding genes or whether particular genes (or respiratory complexes) have undergone changes in evolutionary rates (a scenario that might indicate adaptive or functional changes). Accordingly, we used estimates of site-specific relative variability generated by the SiteVarProt methodology. For each major lineage in our dataset, we both counted the number of amino acid positions that are perfectly conserved within the group and calculated mean gene-specific normalized relative substitution rates for all variable sites (Table 4). Intriguingly, our data show that for proteins that are part of the cytochrome-c oxidase complex (COX1, COX2, COX3) and the cytochrome b protein, the normalized mean relative variability (of variable sites) is higher in Primates/Dermoptera than in Rodentia, while the number of perfectly conserved sites is lower in Primates/Dermoptera. These observations are highly consistent with previous studies that have identified accelerated rates of evolution of nuclear and mitochondrially encoded components of the cytochrome c oxidase complex and cytochrome b in some Primates (eg [31-33]). Conversely, for proteins that are components of complex I (NADH dehydrogenase complex) the mean relative variability of variable sites is somewhat lower in Primates/Dermoptera than in Rodentia while the number of perfectly conserved sites tends to be higher in Rodentia.

**Table 4:** Variability and Numbers of constant sites for Euarchontoglires mitochondrial genes by taxonomic group

	Primates/Dermoptera		Rodentia		Lagomorpha	
Gene	NMVV*	#const**	NMVV*	#const**	NMVV**	#const*
Cox1	0.804	394	0.739	416	0.859	492
Cox2	0.793	119	0.601	148	0.860	206
Cox3	0.874	161	0.815	179	1.076	242
CytB	1.049	207	0.895	246	0.958	323
Atp8	1.486	12	1.246	20	1.092	36
Atp6	1.087	96	0.802	124	1.008	197
Nad1	0.917	159	0.863	161	0.923	271
Nad2	1.095	111	1.291	104	1.039	231
Nad3	1.077	45	1.121	50	0.921	84
Nad4	0.998	214	0.993	206	0.952	352
Nad4l	0.924	44	1.094	40	0.947	71
Nad5	1.012	241	1.113	239	1.076	421

* Normalized Mean Variability for Variable sites, ** Number of constant sites within group

## Discussion

### Protein vs. DNA sequences

The relative merits of performing phylogenetic analyses on nucleotide or corresponding amino acid sequences have been discussed extensively (eg [34]). In brief, while DNA sequences allow the complete parameterization of substitution models through the use of the data under examination, amino acid substitution models typically allow only amino acid frequencies to be adjusted according to the available data. On the other hand, the degree of substitutional saturation and homoplastic character evolution is expected to be higher among nucleotide sequences due to the restricted number of character states and mild to moderate compositional biases in DNA sequences are expected not to cause extensive perturbation of amino acid composition due to the degeneracy of the genetic code, but see [35]. It is clearly desirable that DNA and associated inferred amino acid sequences should generate congruent phylogenetic hypotheses; in the absence of such congruent results it is necessary to assess whether inferences derived from DNA and protein sequences are statistically incongruent and, if so, attempt to explain observed differences in terms of characteristics of the data. In the current investigation, neither dataset discriminates between the two Bayesian consensus trees according to the approximately unbiased test. It is of some concern that the Bayesian consensus tree generated from the DNA data recovers *Anomalurus *not within Rodentia but among Primates. However, we note that the DNA dataset considered includes several primate sequences that fail the chi square test of compositional homogeneity. When Primates are excluded, *Anomalurus *is recovered in an identical position to the amino acid analyses (as sister to the Hystricognathi). Furthermore, while distance bootstrap analyses of protein sequences support, albeit weakly, the monophyly of Rodentia (Fig. 1), equivalent analyses performed on DNA sequences yield poorly supported consensus trees depicting non-monophyletic Glires, Rodentia and Primates/Dermoptera (not shown). Finally, no potential amino-acid synapomorphies link *Anomalurus *with the Primates/Dermoptera clade (while potential synapomorphies with the Hystricognathi and with the Muroidea/Dipodidae clade have been identified). We therefore consider results derived from protein sequences to be more reliable in this case, although we suggest that there is no significant incongruence between inferences derived from the protein and DNA data.

### The phylogeny of Euarchontoglires and the evolutionary placement of *Anomalurus*

Bayesian and distance bootstrap analyses of concatenated first and second codon positions and inferred protein sequences of Rodentia, Primates/Dermoptera, Scandentia and Lagomorpha generated well-supported hypotheses of relationships within Primates/Dermoptera. In accordance with other analyses of mitochondrial sequences [21,6], we recover Primates as paraphyletic with Dermoptera emerging as sister-group to the Anthropoidea with high bootstrap and posterior support. Our protein, but not DNA data reject monophyly of primates as assessed by the AU test of competing tree topologies. Analyses of concatenated nuclear (or nuclear and mitochondrial) data usually (eg [36-38]), but not always [39] prefer the traditional hypothesis of Primates monophyly. However, support for the position of Dermoptera as sister to Scandentia is often scarce and or dependent on the analytical method employed [36]. The positioning of *Tarsius *as sister to *Lemur *and *Nycticebus *is unexpected in the light of morphological and nuclear data, but consistent with other analyses of mt (for discussion see [18]) and some analyses of nuclear data [39,36]. The evolutionary affinities of Scandentia (represented in our analyses by *Tupaia*) have not been satisfactorily resolved by molecular data (see [40,20,39,36,41,21,38] and references therein) although current thinking tends to favour a sister relationship with Dermoptera in a clade which emerges basal to the primates. The analyses of mt protein data presented here are in accord with our previous analyses of mt DNA data [6] in suggesting that *Tupaia *represents the basal divergence of Euarchontoglires rather than constituting the sister taxon of Lagomorpha, Primates, Primates/Dermoptera or Dermoptera. However, where tests of competing tree topologies have been performed, the position of Scandentia has remained unclear [36]. Thus, while our mitochondrial dataset refutes what must be considered a weakly supported nuclear consensus for relationships between Dermoptera, Scandentia and Primates, it is not clear how inconsistent the nuclear data may be with the mitochondrially-derived hypothesis. Importantly, the question of Dermoptera/Primates relationships at least has recently been addressed through examination of the distribution of Short Interspersed Nuclear Elements in these organisms [42]. These data should be free of many of the problems associated with analysis of molecular sequences (substitutional saturation, model choice, compositional bias etc) and strongly support the traditional hypothesis of Primate monophyly – suggesting that available mitochondrial and (to a lesser extent) nuclear sequence data have failed to correctly resolve Primates/Dermoptera relationships.

With respect to relationships within Glires, inferred protein sequences suggested a specific relationship between *Anomalurus *and the Hystricognathi. However, first and second codon positions of the gene sequences tended place *Anomalurus *among the basal divergences in the Primates/Dermoptera clade. This placement was not robustly supported and indeed Bayesian analyses of nucleotide sequences in the absence of Primates (some of whose sequences failed tests of compositional homogeneity) favoured the same placement as suggested by the protein sequences. While we are not aware of published hypotheses suggesting this relationship, it should be noted that a relationship between Anomaluridae and Ctenodactylidae has been proposed on the basis of morphological features [14]. Recent molecular and many classical studies have suggested an affinity between Hystricognathi and Ctenodactylidae (e.g. [11,7,13]). Unfortunately, at the present time, no complete mitochondrial genome sequences from Ctenodactylidae are available. Some molecular data have suggested that the Anomaluridae are specifically related to the Pedetidae (Spring Hares) [12]. Recent analyses that have included sequences from either of these taxa have tended to place these organisms as weakly supported basal branches in a clade containing Dipodidae, Muridae, Geomyidae and Heteromyidae (sister to the Dipodidae/Muroidea clade in our sampling) [36,11,7,43]. Our analyses of constrained tree topologies recovered this placement as a viable alternative to our preferred hypothesis of a relationship between *Anomalurus *and Hystricognathi (and presumably Ctenodactylidae).

With respect to relationships between other families/superfamilies within Rodentia, we consistently recover previously proposed relationships between Dipodidae and Muroidea and between Sciuroidea and Gliridae with high bootstrap and posterior probability support. Our analyses however, like those based on other genes or gene concatenations [39,36,41,11,12,38,7,43] fail to unambiguously resolve relationships between these groups and the Hystricognathi in the sense that high posterior probabilities for higher order relationships within Rodentia are often accompanied by moderate or low bootstrap support and valid probabilistic tests of alternative topologies have seldom been presented. While our data and analyses prefer the hypothesis that the basal divergence within Rodentia consists of Hystricognathi (and by inference Ctenodactylidae) + Anomaluridae, leaving the Dipodidae/Muroidea and Gliridae/Sciuridae clades as sisters to each other, our data do not exclude a multitude of other evolutionary scenarios.

The Slow-Fast method – in which faster evolving sites are progressively removed from the dataset and changes in support for nodes of interest are examined – was employed to investigate whether sites supporting different hypotheses of relationships could be partitioned according to evolutionary rates. Exclusion of fast evolving sites has little impact on the resolution of either the position of *Anomalurus *(when the 25% of sites inferred to be fastest evolving were removed, we recover *Anomalurus *as a weakly supported sister to the Muridae/Dipodidae clade in accordance with constrained topologies discussed previously) or other relationships within Rodentia, suggesting that "noise" from fast evolving sites is not obscuring phylogenetic signal present in slower evolving sites. We interpret this finding as an indication that phylogenetic signal for higher-order relationships within Rodentia is rather scarce. In accordance with this proposal, we observe that the inferred amino acid sequences derived from *Anomalurus *(3519 unambiguously aligned amino acids) share only three potential synapomorphies with the Hystricognathi and three with the Muroidea/Dipodidae clade. There are no potential synapomorphies linking all Rodentia, or associating *Anomalurus *with Lagomorpha, the Myoxidae/Sciuridae clade, Primates/Dermoptera, or any possible sister group set of Rodentia families.

### Molecular dating of divergences in Euarchontoglires

Molecular dating of divergences within Euarchontoglires based on mitochondrial sequence data and a global molecular clock has historically yielded estimates in conflict with the fossil record, particularly with respect to Rodentia e.g. [44,45]. More recently several approaches that allow substitution rates to vary over the tree have been developed (for review see [8]). We have employed a Bayesian relaxed molecular clock approach that does not require the user to specify where rate changes occur on the tree and allows specification of calibration points as intervals rather than fixed dates. Using 6 constraints (upper and lower limits on three nodes) we have generated estimates of divergence times which are highly consistent with estimates of divergence dates based on the fossil record. Notably, the divergence dates recovered for *Homo *vs. *Pan *(5.7 and 6.4MYA for Protein and DNA data respectively), old world vs. new world monkeys (36.8 and 38.5MYA for Protein and DNA data) are highly consistent with both fossil data and other recent molecular dating studies using molecular sequences [46]. We estimate that the divergence of Rodentia occurred 62.8 (protein) or 62.7 (DNA) MYA, the divergence of Hystricognathi + *Anomalurus *occurred 58.8 (protein) or 57.5 (DNA) MYA while the divergence of *Anomalurus *occurred 48.1 (protein) or 52.3 (DNA) MYA and the divergence of the Sciuidae/Myoxidae and Muroidea/Dipodidae clades occurred 53.6 (protein) or 51.8 (DNA) MYA – with the *Mus/Rattus *split occurring 15.1 (protein) or 15.9 (DNA) MYA. These estimates are generally consistent with the fossil data and recent estimates using local clock approaches [13,9,10].

Given the relative lack of resolution of relationships within Rodentia, we were interested to investigate the impact of the tree topology on estimates of divergence dates. Changes in the phylogenetic position of *Anomalurus *yielded relatively minor differences in divergence time estimates. For example, placing Anomalurus as the basal divergence in Rodentia or as sister to the Dipodidae/Muroidea/Myoxidae/Sciuridae group altered estimates of divergence of Rodentia and Lagomorpha by a maximum of 0.12MY. Estimates of the divergence of the Hystricognathi lineage from the Dipodidae/Muroidea/Myoxidae/Sciuridae never varied by more than 3.5MY and the divergence of *Anomalurus *by at most 2.8MY (not shown).

These findings are notable as they highlight a fundamental problem in the resolution of higher order relationships within Rodentia. Accounting for the 5% error intervals of our dating estimates, the divergence of Rodentia from Lagomorpha, the divergence of Hystricognathi from other Rodentia and the divergence of Sciuridae/Myoxidae and Muroidea/Dipodidae potentially occurred within 3.1 million years of each other around 60 million years ago – leaving relatively little time for the evolution of lineage-specific characters (molecular or morphological) which may be used in the reconstruction of phylogenetic affinities. Conversely, the relatively long subsequent independent evolutionary history of lineages considered here, in conjunction with the limited available taxonomic sampling is likely to have lead to extensive symplesiomorphy and homoplasy, further complicating phylogenetic reconstruction.

## Conclusion

The use of mitochondrial sequences for the investigation of even relatively shallow phylogenetic relationships within Rodentia has recently been questioned [47,48]. Indeed it has long been suspected that fast evolutionary rates and compositional biases can lead to misleading phylogenetic signal and poorly supported splits for deeper relationships. While we agree that saturation and compositional biases present a major problem for the reconstruction of ancient divergences, we stress that conclusions from mitochondrial sequences regarding divergence times are consistent with fossil data. Indeed recent studies using individual and concatenated nuclear or nuclear and mitochondrial gene sequences also fail to robustly resolve higher-level relationships within Rodentia [36,11,12,38]. Given the aforementioned considerations, we suggest that difficulties in the reconstruction of correct and unambiguous higher-order relationships within Glires do not reflect limitations of either nuclear or mitochondrial sequence data, but are likely to be inherent consequences of a rapid evolutionary radiation which occurred around 60 million years ago.

## Methods

### DNA extraction, amplification and sequencing

Mitochondrial DNA was extracted from 4.5 g of frozen liver of an *Anomalurus sp *(scaly-tailed flying squirrel) specimen captured in central Africa (specimen provided by F. Catzeflis), according to previously described methods for mammalian species [49].

The entire mitochondrial genome was amplified, using the Polymerase Chain Reaction with eight pairs of heterologous primers designed on the basis of highly conserved regions of the complete mitochondrial sequence of several representative species mammalian species [6]. Amplifications were performed in 100 μl reaction volumes containing 10 mM Tris-HCl (pH 8.3), 50 mM KCl, 1.5 mM MgCl_2_, 0.001% (w/v) gelatin, 0.25 mM of each dNTP, 0.5 μM of each primer, and 2.5U of TaqGold polymerase (Roche Applied Science). PCR cycling conditions were 10 min of hot start at 95°C for the activation of the enzyme, followed by 30–35 amplification cycles (45 s of denaturation at 95°C, 45 s of primer annealing at temperatures from 55 to 65°C, and 2 to 3 min of extension at 72°C) followed by a final cycle of 7 min at 72°C. Single amplification products with length between 1.2 and 3.5 Kb were consistently obtained and produced overlapping fragments that covered the whole mitochondrial genome. PCR products were purified using the Amicon Microcon-PCR Centrifugal Filter Devises (Millipore) following the manufacturer's instructions. Fragments were sequenced either directly or after cloning in the pGEM-t easy vector (Promega). Sequencing reactions were performed using the Thermo Sequenase Cy5.5 Dye Terminator Cycle Sequencing Kit (Amersham Pharmacia Biotech) in 8 μl reaction volumes and following the manufacturer's instructions. After purification, DNA sequences were analyzed on a Seq4×4 automated sequencer (Amersham Pharmacia Biotech). Double strand primer walking strategy provided contiguous sequence information for both strands in all fragments. All overlapping regions between amplified fragments matched perfectly and all predicted open reading frames followed the vertebrate mitochondrial genetic code, leading us to exclude the possibility that we had amplified fragments of mitochondrial genome that had been inserted into the nuclear genome. The mtDNA sequence of the flying squirrel *Anomalurus sp*. has a G+C content of 46.16% and has been deposited in the EMBL database under the accession number AM_159537.

### Phylogenetic analyses

Conceptually translated coding sequences H-strand genes derived from all available complete mitochondrial genomes of Primates, Dermoptera, Scandentia, Rodentia and Lagomorpha species were aligned using the program MUSCLE [50] [see Additional file 1]. Sequences from the Laurasiatheria species sheep, dog and mole were included as outgroups (a total of 41 taxa, see table included in supplementary materials). Alignments were manually adjusted and DNA sequences reverse aligned to correspond with protein alignments. Regions of low alignment quality were identified using the program G-Blocks [51] and excluded from subsequent analyses. Protein sequences and the ungapped first and second codon positions (after exclusion of codons with first position leucine synonymous substitutions (Leu-SynP1)) of DNA sequences, were included in concatenated datasets for phylogenetic analyses (5358 nucleotides, 3519 amino acids).

Phylogenetic analyses were carried out using the program MrBayes 3.1 [52] using the General-Time-Reversible (GTR) substitution model for nucleotide sequences and "mtrev24" model for protein sequences, in both cases with the invariant site plus gamma options (eight categories). Two parallel analyses, each composed of one cold and three incrementally heated chains were run for 2,000,000 generations. Trees were sampled every 50 generations and 20,000 trees were discarded as "burn-in" (sufficient to allow convergence according to the tests indicated by the program).

Distance bootstrap analyses were performed by using the shellscript PUZZLEBOOT (available from the TREE_PUZZLE website) in conjunction with TREE-PUZZLE [17] and the programs SEQBOOT, NEIGHBOR and CONSENSE from the PHYLIP package [53], using the substitution models employed in Bayesian analyses with rate heterogeneity parameters estimated by TREE-PUZZLE on the relevant Bayesian tree topology.

For tests of alternative tree topologies, site likelihoods were calculated under the GTR + gamma and mtrev24 + gamma models (for DNA and protein data respectively) using the PAML package [54]. The Approximately Unbiased (AU) tests were performed using the software CONSEL [19].

Bayesian relaxed molecular clock dating analyses were performed using the MULTIDISTRIBUTE package [24] in conjunction with programs from the package PAML. For DNA sequences, the F85 + gamma model (the most complex model available in BASEML) was employed. For protein sequences, following the method of Amer and Kumazawa [25], a modified version of CODEML was used to estimate model parameters for the mtrev24 + gamma model. In both cases the program ESTBRANCHES [24] was used to estimate variances of branch lengths and MULTIDIVTIME [24] used to estimate divergence times.

Analyses of compositional homogeneity were performed using the Chi square test implemented in the program TREE-PUZZLE. Site-specific relative substitution rates were estimated using the SiteVarProt algorithm [23].

## Authors' contributions

DSH performed most of the phylogenetic and dating analyses and drafted the manuscript. KL sequenced the mt genome of *Anomalurus*. AR isolated genomic DNA, assembled contigs, annotated the genome sequence and devised cloning and sequencing strategies. CG constructed sequence alignments, performed some phylogenetic analyses and helped draft the manuscript. CS conceived the project and helped draft the manuscript. GP participated in design and coordination of the study, performed some statistical analyses and helped to draft the manuscript. All authors read and approved the final manuscript

## Supplementary Material

Additional file 1 **List of sequences/taxa utilized**. A pdf file containing classification, accession numbers and common names of sequences/organisms used in the current study Click here for file
