# Supplementary material for: Phylogenetic analyses of complete mitochondrial genome sequences suggest a basal divergence of the enigmatic rodent Anomalurus
Source: BMC Evol Biol. 2007 Feb 8;7:16. doi: 10.1186/1471-2148-7-16 (PMC1802082; doi:10.1186/1471-2148-7-16)
Supplement: Additional file 1 — List of sequences/taxa utilized. A pdf file containing classification, accession numbers and common names of sequences/organisms used in the current study [file 1471-2148-7-16-S1.pdf]

Organism	Accession	classification	Common name
Anomalurus sp.	AM159537	Rodentia / Anomaluridae	Scaly tailed flying squirrel
Canis familiaris	NC_002008	Laurasiatheria OUTGROUP	dog
Cavia porcellus	NC_000884	Rodentia / Hystricognathi	domestic guinea pig
Cebus albifrons	NC_002763	Primates / Haplorrhini	white-fronted capuchin
Chlorocebus aethiops	NC_007009	Primates / Haplorrhini	African green monkey
Chlorocebus sabaeus	NC_008066	Primates / Haplorrhini	green monkey
Colobus guereza	NC_006901	Primates / Haplorrhini	guereza
Cricetulus griseus	NC_007936	Rodentia / Sciurognathi / Muroidea	Chinese hamster
Cynocephalus variegatus	NC_004031	Dermoptera	Malayan flying lemur
Cynocephalus variegatus	AJ428849	Dermoptera	Malayan flying lemur
Gorilla gorilla	NC_001645	Primates / Haplorrhini	gorilla
Homo sapiens	NC_001807	Primates / Haplorrhini	human
Hylobates lar	NC_002082	Primates / Haplorrhini	common gibbon
Jaculus jaculus	NC_005314	Rodentia / Sciurognathi / Dipodidae	lesser Egyptian jerboa
Lemur catta	NC_004025	Primates / Strepsirrhini	ring-tailed lemur
Lepus europaeus	NC_004028	Lagomorpha	European hare
Macaca mulatta	NC_005943	Primates / Haplorrhini	rhesus monkey
Macaca sylvanus	NC_002764	Primates / Haplorrhini	Barbary ape
Microtus kikuchii	NC_003041	Rodentia / Sciurognathi / Muroidea	Taiwan vole
Mus musculus	NC_005089	Rodentia / Sciurognathi / Muroidea	house mouse
Mus musculus domesticus	NC_006914	Rodentia / Sciurognathi / Muroidea	western European house mouse
Mus musculus molossinus	NC_006915	Rodentia / Sciurognathi / Muroidea	Japanese wild mouse
Myoxus glis	NC_001892	Rodentia / Sciurognathi / Myoxidae	fat dormouse
Nannospalax ehrenbergi	NC_005315	Rodentia / Sciurognathi / Muroidea	Ehrenberg's mole-rat
Nycticebus coucang	NC_002765	Primates / Strepsirrhini /	slow loris
Ochotona collaris	NC_003033	Lagomorpha	collared pika
Ochotona princeps	NC_005358	Lagomorpha	American pika
Oryctolagus cuniculus	NC_001913	Lagomorpha	rabbit
Ovis aries	NC_001941	Laurasiatheria OUTGROUP	sheep
Pan paniscus	NC_001644	Primates / Haplorrhini	pygmy chimpanzee
Pan troglodytes	NC_001643	Primates / Haplorrhini	chimpanzee
Papio hamadryas	NC_001992	Primates / Haplorrhini	hamadryas baboon
Pongo pygmaeus	NC_001646	Primates / Haplorrhini	orangutan
Pongo pygmaeus abelii	NC_002083	Primates / Haplorrhini	Sumatran orangutan
Rattus norvegicus	NC_001665	Rodentia / Sciurognathi / Muroidea	Norway rat
Sciurus vulgaris	NC_002369	Rodentia / Sciurognathi / Sciuridae	Eurasian red squirrel
Talpa europaea	NC_002391	Laurasiatheria OUTGROUP	European mole
Tarsius bancanus	NC_002811	Primates / Haplorrhini	western tarsier
Thryonomys swinderianus	NC_002658	Rodentia / Hystricognathi	greater cane rat
Trachypithecus obscurus	NC_006900	Primates / Haplorrhini	dusky leaf monkey
Tupaia belangeri	NC_002521	Scandentia / Tupaiidae	northern tree shrew
